# Sex-Related Differences in Mortality, Delayed Cerebral Ischemia, and Functional Outcomes in Patients with Aneurysmal Subarachnoid Hemorrhage: A Systematic Review and Meta-Analysis

**DOI:** 10.3390/jcm13102781

**Published:** 2024-05-09

**Authors:** Sarah Berli, Massimo Barbagallo, Emanuela Keller, Giuseppe Esposito, Alberto Pagnamenta, Giovanna Brandi

**Affiliations:** 1 Faculty of Medicine, University of Zurich, 8032 Zurich, Switzerland; 2 Neurocritical Care Unit, Department of Neurosurgery, Institute for Intensive Care Medicine, University Hospital Zurich, 8091 Zurich, Switzerland; 3 Clinical Neuroscience Center, University Hospital Zurich and University of Zurich, 8091 Zurich, Switzerland; 4 Department of Neurosurgery, University Hospital Zurich, 8091 Zurich, Switzerland; 5 Clinical Trial Unit, Ente Ospedaliero Cantonale, 6900 Lugano, Switzerland; 6 Department of Intensive Care, Ente Ospedaliero Cantonale, 6900 Lugano, Switzerland; 7 Division of Pneumology, University of Geneva, 1211 Geneva, Switzerland

**Keywords:** sex differences, aneurysmal subarachnoid hemorrhage, mortality, delayed cerebral ischemia, functional outcome

## Abstract

**Background/Objective**: Sex-related differences among patients with aneurysmal subarachnoid hemorrhage (aSAH) and their potential clinical implications have been insufficiently investigated. To address this knowledge gap, we conduct a comprehensive systematic review and meta-analysis. **Methods**: Sex-specific differences in patients with aSAH, including mortality, delayed cerebral ischemia (DCI), and functional outcomes were assessed. The functional outcome was dichotomized into favorable or unfavorable based on the modified Rankin Scale (mRS), Glasgow Outcome Scale (GOS), and Glasgow Outcome Scale Extended (GOSE). **Results**: Overall, 2823 studies were identified in EMBASE, MEDLINE, PubMed, and by manual search on 14 February 2024. After an initial assessment, 74 studies were included in the meta-analysis. In the analysis of mortality, including 18,534 aSAH patients, no statistically significant differences could be detected (risk ratio (RR) 0.99; 95% CI, 0.90–1.09; *p* = 0.91). In contrast, the risk analysis for DCI, including 23,864 aSAH patients, showed an 11% relative risk reduction in DCI in males versus females (RR, 0.89; 95% CI, 0.81–0.97; *p* = 0.01). The functional outcome analysis (favorable vs. unfavorable), including 7739 aSAH patients, showed a tendency towards better functional outcomes in men than women; however, this did not reach statistical significance (RR, 1.02; 95% CI, 0.98–1.07; *p* = 0.34). **Conclusions**: In conclusion, the available data suggest that sex/gender may play a significant role in the risk of DCI in patients with aSAH, emphasizing the need for sex-specific management strategies.

## 1. Introduction

Subarachnoid hemorrhage (SAH) is a type of stroke that accounts for approximately 5% of all strokes [1]. Despite its low frequency, it is associated with high mortality and morbidity rates, including long-term cognitive impairment and reduced quality of life [2]. Aneurysmal subarachnoid hemorrhage (aSAH) is responsible for 85% of nontraumatic SAH cases and occurs when an aneurysm ruptures [1]. Since patients with aSAH are usually younger than patients with ischemic stroke and are still workers at the time of the bleeding, aSAH represents a global economic burden on society and patients [3].

Sex-associated differences in the epidemiology of aSAH are well known. Females suffer more frequently from aSAH than men at all ages [4]. Some possible reasons for this include the vulnerability of the walls of the blood vessels, the interference of collagen and elastin, and hormonal influences that may contribute to the formation of aneurysms in women [5,6]. Additionally, some risk factors, such as smoking, can increase the likelihood of aneurysm rupture more significantly in women [7,8]. Furthermore, differences in aneurysm location have been described. Men are more likely to have aneurysms in the anterior cerebral artery, while in females, aneurysms are mainly located along the internal carotid artery [9,10,11].

On the other hand, less is known about sex differences in the frequency of delayed cerebral ischemia (DCI), functional outcomes, and mortality in aSAH patients. The results of the available literature, in fact, are often contradictory [12,13,14]. Some studies have found that being female is associated with a higher risk of poor outcomes following aSAH, including a higher 30-day case-fatality rate [15] and poorer 2-year outcomes compared to men [16,17,18]. However, other studies have reported that sex is not a determining factor for outcome following aSAH [12,19,20]. DCI is a common complication that occurs in almost 30% of patients after aSAH [21], and it is a significant predictor of unfavorable outcomes [22,23]. While some studies have suggested that women are more likely to experience DCI than men [14,24], others have concluded that there is limited evidence of a sex difference [25].

Understanding the relationship between sex and mortality, DCI, and functional outcomes in patients with aSAH is crucial to developing appropriate and personalized interventions. For example, the identification of patients at higher risk for complications, such as DCI, could have consequences for resource utilization, such as the frequency of clinical and radiological controls to prevent and detect them early.

Hence, we performed a systematic review and meta-analysis of existing data focusing on sex-related differences in mortality, frequency of DCI, and functional outcomes in patients with aSAH.

## 2. Methods

PRISMA guidelines (Preferred Reporting of Items in Systematic Reviews and Meta-analyses) were employed to guide review processes [26]. This systematic review was registered at The International Prospective Register of Systematic Reviews (PROSPERO; registration number CRD42024508960).

### 2.1. Search Strategy

Studies were identified in the MEDLINE, EMBASE, and PubMed electronic databases. The search was performed on 14 February 2024. The search strategy combined three concepts: (1) gender, sex, sex difference, sex ratio, women, men, female, and male, as well as Medical Subject Heading related; (2) terms related to critical care (i.e., intensive care, intensive care unit, ICU, critically ill patient), and terms related to our outcomes of interest (i.e., mortality, delayed cerebral ischemia, modified Rankin Scale, Glasgow Outcome Scale, Glasgow Outcome Scale Extended), as well as Medical Subject Heading related; and (3) terms related to aneurysmal subarachnoid hemorrhage (i.e., subarachnoid hemorrhage, brain hemorrhage, brain bleeding, brain artery aneurysm rupture, hemorrhagic stroke), as well as Medical Subject Heading related.

The electronic search was supplemented by a manual search of reference lists and recent reviews.

Two reviewers (SB and GB) independently screened titles and abstracts using the platform Covidence. Duplicates were excluded. Secondly, they screened the corresponding publications in full text to assess if the studies met the inclusion criteria. The software notified the reviewers if there were discrepancies, and they were solved through discussion. If consensus could not be reached, a third reviewer mediated to resolve the conflict.

### 2.2. Inclusion and Exclusion Criteria

The study selection criteria are presented in Table 1 using the PICOS (Population, Interventions, Comparisons, Outcomes, and Study Design) acronym.

All identified studies were reported using a flowchart according to PRISMA guidelines.

Studies in adult patients with aSAH, which included any of the following outcomes of interest, were considered for eligibility: mortality, DCI, and/or functional outcomes. We assessed the most commonly used functional outcomes in aSAH clinical trials [27], including the modified Rankin Scale (mRS) [28,29], the Glasgow Outcome Scale (GOS) [30], and the Glasgow Outcome Scale Extended (GOSE) [31,32].

Based on the mRS, GOS, and GOSE, the functional outcome was dichotomized into “favorable” or “unfavorable”. A favorable outcome was defined as mRS 0–2, GOS 4–5, and GOSE 5–8. In many clinical trials and in most of the studies included in this analysis (15 of 18 studies), the functional outcome is dichotomized for the analysis into “favorable” and “unfavorable”. Considering the variability in studies regarding the score classified as a favorable or unfavorable outcome in the mRS, we referred to the definition of the European Stroke Organization, where an mRS score of 0–2 is considered favorable [33].

For our analysis, we used only those studies that defined DCI as clinical deterioration (a new focal neurologic deficit or decrease in level of consciousness) deemed secondary to vasospasm and/or a new cerebral infarct after excluding other possible causes [34,35]. The included studies employed various methods to detect vasospasm, such as computerized tomography angiography, a magnetic resonance perfusion scan, transcranial Doppler, or digital subtraction angiography. Cerebral infarction was determined using computed tomography or magnetic resonance imaging. We excluded from our analysis studies that considered asymptomatic vasospasm as a part of their definition of DCI.

Randomized controlled trials (RCTs), prospective observational studies, and retrospective studies with more than 10 patients and clinical registries were eligible. Only studies written in English were included and published from the year 2000 onwards.

Studies that did not distinguish outcomes according to sex or did not include any of the outcomes of interest were not considered eligible. Case reports, case series with less than 10 patients, animal studies, abstracts, and reports with no values in the results were excluded.

### 2.3. Data Extraction 

The trial’s characteristics (first author and publication year); type of study; the number of patients included; the number of females/males; selected outcome results (mortality, DCI, functional outcomes, as assessed with the mRS, GOS, or GOSE); and time of measurement were extracted and summarized using a pre-defined Excel Table.

### 2.4. Statistical Analysis

We calculated a pooled estimate of the risk ratio (RR) with a 95% confidence interval (95%-CI) for each dichotomous outcome (mortality, DCI, dichotomized functional outcomes (favorable/unfavorable)) by sex. The decision to calculate the RR was driven by the dichotomous nature of the three outcomes and the high proportion of cohort studies in our analysis. Additionally, the RR has the advantage of being easier to interpret compared to the odds ratio [36,37,38]. Each trial-specific effect size was subsequently combined across studies in order to calculate summary estimates and presented as a forest plot. We evaluated heterogeneity by the chi-squared test and calculated I^2^ [39]. To further estimate the effect size of future studies with similar settings, we applied prediction intervals [40]. The Mantel–Haenszel test was used to construct the random effects model [41].

The presence of publication bias was explored with a funnel plot. All statistical analyses were performed using R, version 4.3.0 [42].

## 3. Results

**Figure 1:**
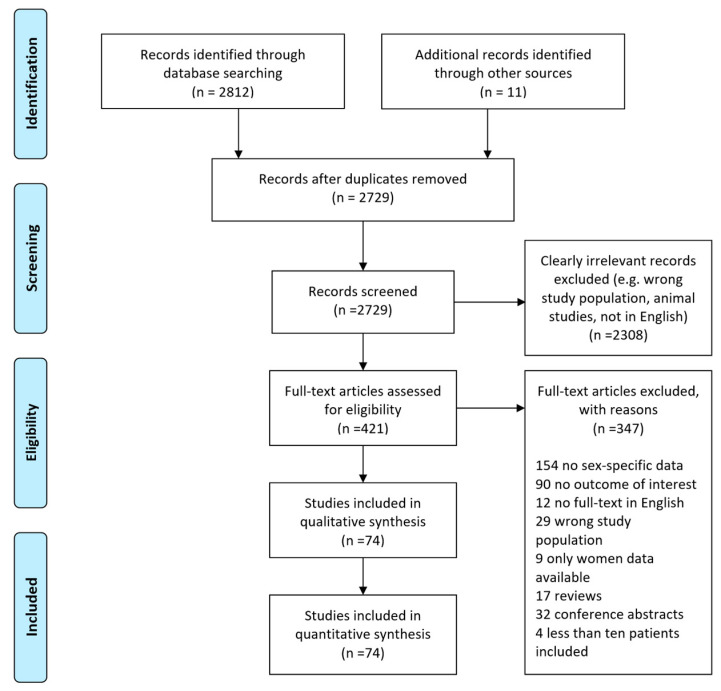
Study selection flow diagram according to PRISMA guidelines.

The systematic search identified 2812 studies. By manual search, 11 additional studies were identified. Overall, 2823 abstracts were considered as potentially eligible. After screening based on the inclusion criteria, 421 were selected for full-text review (see flow diagram in Figure 1). Finally, a total of 74 studies were included in the quantitative synthesis (meta-analysis).

The list and characteristics of the included studies are presented in Supplementary Tables S1–S3.

### 3.1. Mortality

Nineteen studies, referring to 18,534 patients with aSAH, were included in the meta-analysis (with 7067 male and 11,467 female patients) [12,13,43,44,45,46,47,48,49,50,51,52,53,54,55,56,57,58,59]. Mortality was evaluated at different time points, from in-hospital mortality/mortality at the intensive care unit (ICU) to 12 months after aSAH. However, most of the studies evaluated mortality as in-hospital mortality or at 30 days (12 of 19 studies). The included studies were published between 2002 and 2023.

**Figure 2:**
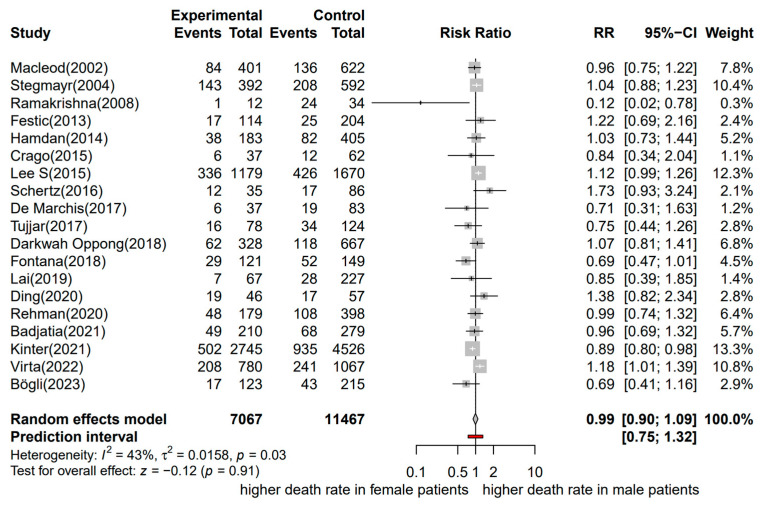
Forest plot of mortality in patients with aSAH analyzed by sex. The risk ratio for mortality at the end of follow-up. Comparison between male and female patients with aneurysmal subarachnoid hemorrhage [12,13,43,44,45,46,47,48,49,50,51,52,53,54,55,56,57,58,59]. CI, confidence interval; RR, risk ratio.

**Figure 3:**
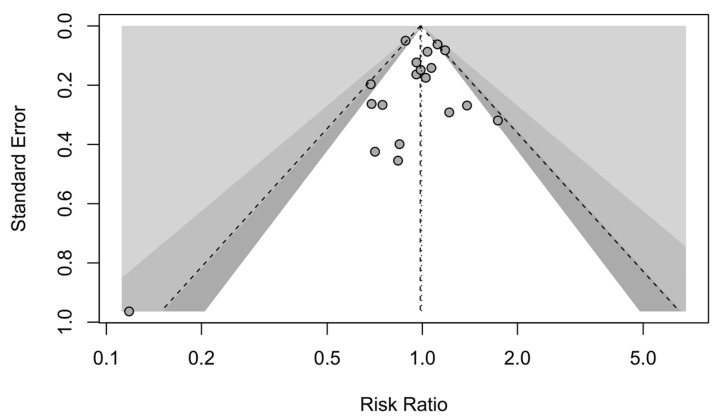
Contour-enhanced funnel plot. Mortality studies with contour levels of 0.9, 0.95, and 0.99, respectively.

A forest plot of the stratified analysis showed no statistically significant difference in mortality between male and female patients with aSAH (RR, 0.99; 95% CI, 0.90–1.09; *p* = 0.91). Heterogeneity testing showed I^2^ = 43% and *p* = 0.03. The prediction interval ranged from *g* = 0.75 to 1.32 (see Figure 2). The assessment of publication bias using a contour-enhanced funnel plot indicated symmetry, and most of the data corresponded to points within the 95% CI, as shown in Figure 3.

### 3.2. Delayed Cerebral Ischemia

Fifty-five studies including 23,864 patients with aSAH were considered in the meta-analysis with DCI as the outcome of interest (8126 males and 15,738 females) [12,13,14,24,44,45,46,49,54,59,60,61,62,63,64,65,66,67,68,69,70,71,72,73,74,75,76,77,78,79,80,81,82,83,84,85,86,87,88,89,90,91,92,93,94,95,96,97,98,99,100,101,102,103,104]. These studies were published between 2000 and 2023.

**Figure 4:**
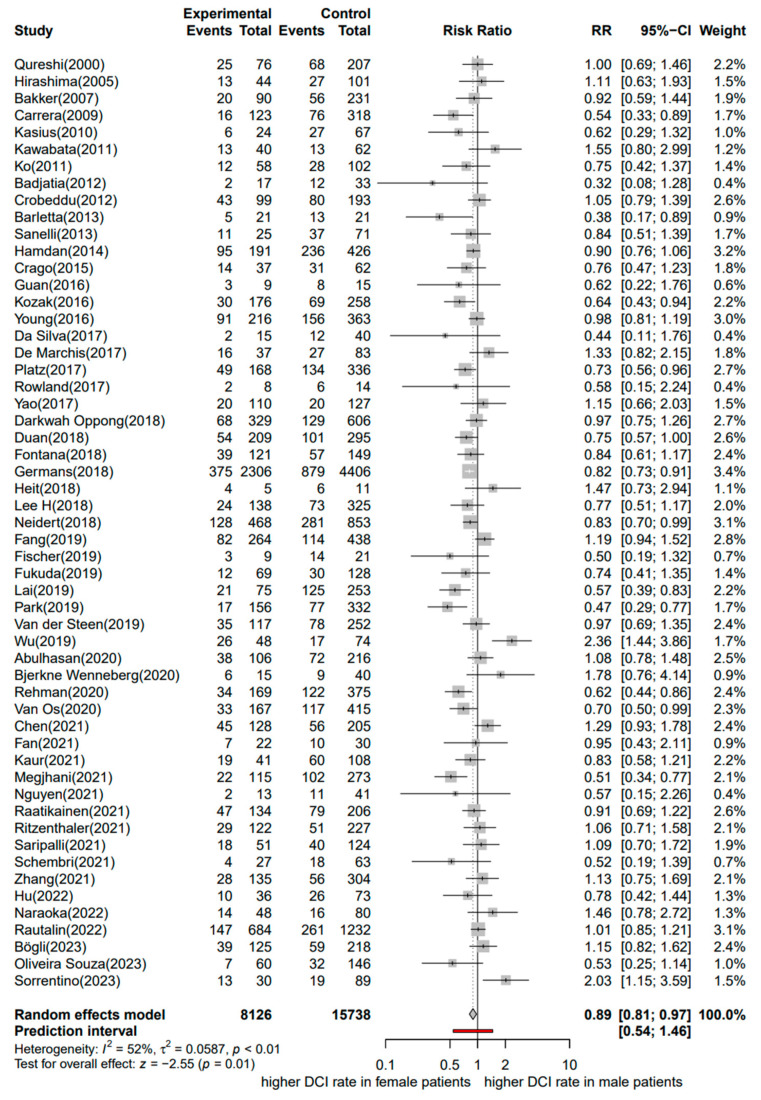
Forest plot of DCI in patients with aSAH analyzed by sex. The risk ratio for DCI. Comparison between male and female patients with aneurysmal subarachnoid hemorrhage [12,13,14,24,44,45,46,49,54,59,60,61,62,63,64,65,66,67,68,69,70,71,72,73,74,75,76,77,78,79,80,81,82,83,84,85,86,87,88,89,90,91,92,93,94,95,96,97,98,99,100,101,102,103,104]. CI, confidence interval; DCI, delayed cerebral ischemia; RR, risk ratio.

**Figure 5:**
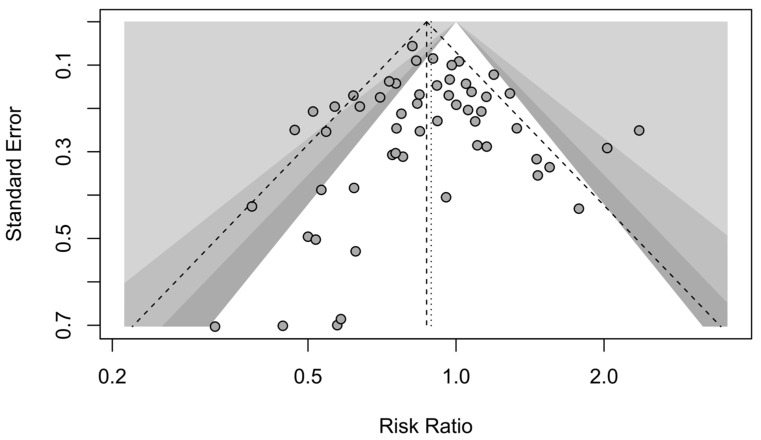
Contour-enhanced funnel plot. DCI studies with contour levels of 0.9, 0.95, and 0.99, respectively.

A forest plot of the stratified analysis demonstrated a significantly higher risk for DCI in females than in males after aSAH. Male patients had a 0.89-fold lower risk of DCI than female patients (11% relative risk reduction; RR, 0.89; 95% CI, 0.81–0.97; *p* = 0.01). Heterogeneity testing showed I^2^ = 52% and *p* < 0.01. The prediction interval ranged from *g* = 0.54 to 1.46 (see Figure 4). The assessment of publication bias using a contour-enhanced funnel plot indicated asymmetry, and most of the data corresponded to points within the 95% CI, as shown in Figure 5. Asymmetry suggests the possibility of either publication bias or a systematic difference between studies of higher and lower precision.

### 3.3. Functional Outcomes

Eighteen studies, including 7739 patients with aSAH, were considered in the meta-analysis with the functional outcome as the outcome of interest (2694 were males and 5045 females) [12,13,44,45,46,47,49,57,59,103,105,106,107,108,109,110,111,112]. The functional outcome was assessed using the mRS in 11 studies, including 3380 patients. The GOS was used in six studies, including 4021 patients, and the GOSE was used in one study, including 338 patients. The time of assessment of the functional outcome varied from hospital discharge to 18 months. However, in most of the studies, the functional outcome was assessed at 3 to 6 months after aSAH (12 of 18 studies). The included studies were published between 2007 and 2023.

**Figure 6:**
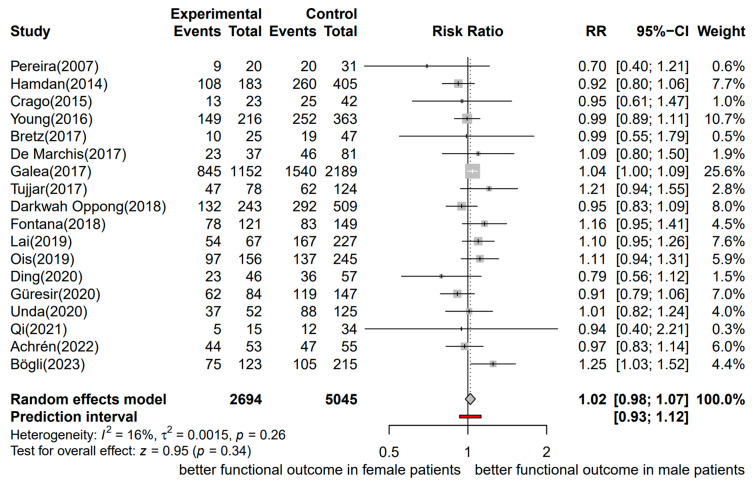
Forest plot of the functional outcome in patients with aSAH analyzed by sex, assessed by the mRS, GOS, and GOSE. The risk ratio for functional outcomes. Comparison between male and female patients with aneurysmal subarachnoid hemorrhage [12,13,44,45,46,47,49,57,59,103,105,106,107,108,109,110,111,112]. CI, confidence interval; RR, risk ratio.

**Figure 7:**
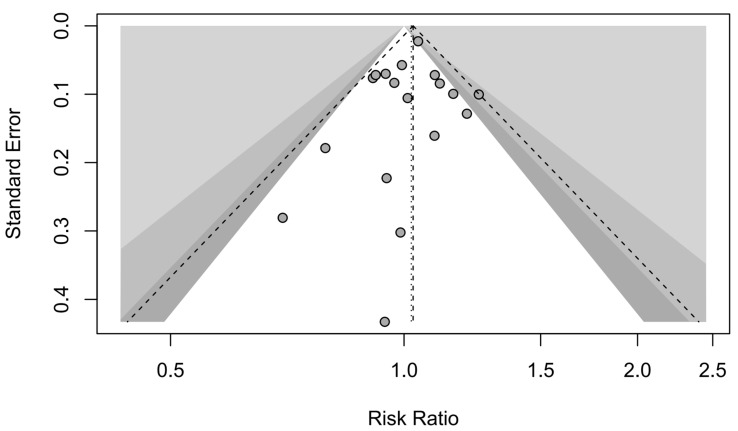
Contour-enhanced funnel plot. Functional outcome studies with contour levels of 0.9, 0.95, and 0.99, respectively.

A forest plot of the stratified analysis showed a trend for a better dichotomized functional outcome in male patients versus female patients after aSAH; however, this was not statistically significant (RR, 1.02; 95% CI, 0.98–1.07; *p* = 0.34). Heterogeneity testing showed I^2^ = 16% and *p* = 0.26. The prediction interval ranged from *g* = 0.93 to 1.12 (see Figure 6). The assessment of publication bias using a funnel plot indicated asymmetry, and all the data corresponded to points within the 95% CI, as shown in Figure 7. Asymmetry suggests the possibility of either publication bias or a systematic difference between studies of higher and lower precision.

## 4. Discussion

We conducted a systematic review and meta-analysis to update the available evidence on sex-related differences in mortality, risk of DCI, and functional outcomes in patients with aSAH. Overall, 74 studies were included in the analysis, including a sample of 18,534 patients for the outcome mortality, 23,864 patients for DCI, and 7739 for functional outcomes. According to our results, we found that the mortality risk after aSAH is similar in females and males. In terms of functional outcomes, males showed a tendency towards better outcomes than females; however, they did not reach statistical significance. Interestingly, males had a significantly lower risk of developing DCI than females.

### 4.1. Results in Context

To our knowledge, our systematic review is the first one that evaluates at the same time the sex differences in risk of DCI, functional outcomes, and mortality in patients with aSAH. This was performed to improve the characterization of these outcomes and assess their relevance since patients with DCI have a higher risk of mortality [113] and worse outcomes [114]. Interestingly, contrary to expectations, despite women having a higher risk of DCI, no higher mortality or significantly worse functional outcomes were found in women.

Recently, Rehman et al. performed a systematic review and meta-analysis to investigate if sex is a predictor for DCI in patients with aSAH [54]. In contrast to this previous work, we did not consider asymptomatic vasospasm as DCI; instead, we included only those studies that defined DCI as a clinical deterioration deemed secondary to vasospasm and/or a new cerebral infarct after excluding other possible causes [34,35]. In our opinion, this choice makes the comparisons among studies more homogenous. Additionally, it is important to note that asymptomatic vasospasm does not have the same negative impact on outcomes as symptomatic vasospasm [115], so its relevance to clinical practice may not be as significant. Nevertheless, consistent with the previous findings, we found a higher risk for DCI among women than men. This higher risk for DCI development in women could be a factor to consider for more efficient utilization of resources, which could imply the need for differentiated management where women may require more intensive neuromonitoring and more frequent radiological controls to prevent and detect DCI.

The reason why women are more likely to develop DCI than men is not completely understood. Contrary to the results of experimental studies [116,117], a possible effect of the sexual hormones on the Doppler blood flow velocities in cerebral vessels in patients with aSAH could not be demonstrated [118]. Furthermore, sex-related differences in the management and delivery of care of patients with aSAH to the disadvantage of women—as already shown in other medical conditions—could play a role in the development of DCI, and these should be further investigated [119,120,121].

Considering mortality and functional outcomes, we did not find significant differences between women and men. Some considerations are needed to interpret these findings. Women are generally older when they suffer from aSAH [122,123], and older age is well-known as a determinant for poor clinical outcomes in patients with aSAH [124,125]. Only based on this, one might expect a higher mortality and a worse functional outcome for women. On the other hand, however, we do not know whether women and men in the study population had the same severity of aSAH and the same intensity of treatment. In addition, patients with aSAH often die after a redirection of care to palliation. This could be another factor since sex-related differences in frequency and the kind of limitations of life-sustaining therapies have already been reported [126]. In non-neuro-intensive care settings, the female sex has been found to be associated with a higher likelihood of limitation of life-sustaining therapies [127]. Furthermore, men are more likely to receive intensive care at the end of life, while women are more likely to state a preference for the limitation of life-sustaining therapies [128,129,130,131]. Given that the causes of death were not present in most of the included publications, we are unable to give a solid explanation for the relation between mortality and sex in patients with aSAH.

Since most of the identified studies evaluated the functional outcome in a dichotomized way (favorable vs. unfavorable), we also decided to maintain the same dichotomization in our analysis. However, analyses of trials using such dichotomous approaches could result in a loss of information and a risk of ignoring bi-directional effects, and they often require larger samples than ordinal approaches [132,133,134]. Despite these pitfalls of dichotomization, ordinal analyses continue to be poorly adopted [135,136], and dichotomous approaches continue to be favored as the primary outcome by many high-profile trials [137,138]. One reason for this may be the poor clinical interpretability of conventional ordinal approaches, which provide outputs, like *p*-values or standard odds ratios, without intuitive effect sizes [139].

We quantified statistical heterogeneity using prediction intervals, in addition to the chi-squared test, and calculated I². Prediction intervals also offer an estimate of where the true effects can be expected for future studies with similar characteristics [40]. Our analysis found that for mortality, DCI, and functional outcomes, the prediction intervals contained the null effect value, indicating that sex may not be a significant factor in some situations [140,141].

### 4.2. Implications for Practice and for Research

Our research highlights a higher risk of DCI in women, which could imply a need for stricter monitoring at hospitals in those patients, including serial daily transcranial Doppler measurements and the insertion of multimodal neuromonitoring for the early detection of DCI. On the other hand, men with lower risk might require less frequent monitoring of DCI in terms of imaging or be transferred to a regular ward bed or discharged more quickly than women. Therefore, understanding the differences between male and female patients with aSAH can help optimize intensive care resources, as demand often exceeds supply. Furthermore, the early detection of DCI in women can have a positive impact on patients and the community, as DCI patients are often discharged to rehabilitation due to their worse functional status [114], which could lead to increased costs for the health system.

Although our study did not evaluate sex differences between cardiovascular risk factors, it is important to note that studies have shown that women are more severely impacted by cardiovascular risk factors [7]. For instance, smoking has a three-fold higher impact on women than men in the development of aSAH [8]. Therefore, it may be necessary to intensify care in the preventive and educational management of risk factors in women.

In addition, previous studies have suggested that there might be an unconscious gender bias in critical care units, which could lead to less aggressive treatment for women [126]. This bias has been also observed in critically ill patients with cardio- and neurovascular diseases in a large nationwide cohort in Switzerland, where women were less likely to receive ICU treatment, regardless of the severity of their condition [120]. Therefore, it is crucial for intensivists and emergency physicians to carefully reassess whether critically ill women are at risk of not receiving adequate care. To ensure equal application of intensive therapy, we need to address gender biases in algorithms of triage and local protocols. Thus, including a standardized protocol for patients with aSAH and an interdisciplinary approach in neurocritical care units could help minimize the impact of any potential gender bias on medical decisions [142].

In terms of research, as new information is continuously discovered regarding the relationship between sex and aneurysm/SAH outcomes, it is important for studies to include sex as a predictor variable in their analyses and examine sex-specific effects of interventions. This is especially important in randomized trials of medical therapies and interventions, where biologically significant relationships might exist between sex and interventions [9].

Regarding DCI, further research should focus on identifying the factors responsible for its development in women, such as vascular structure, hormones, or genetics. To establish sex-specific management guidelines for aneurysmal subarachnoid hemorrhage (aSAH), there is a need for prospective studies and clinical trials to examine sex differences in aSAH management and outcomes, including complications.

### 4.3. Limitations/Strengths

This review has some limitations that should be considered while interpreting the findings. First, this review mainly included retrospective studies. Therefore, there could be some missing data or unadjusted data that might have affected the analysis. Second, the outcomes of interest in the included studies were evaluated at different follow-up times, ranging from ICU/hospital discharge to 18 months. Third, there was low to moderate heterogeneity in some outcomes, which could be due to differences in baseline characteristics between males and females along with variations in sample sizes. Fourth, sex-related differences in risk factors, such as smoking and hypertension, for aSAH could not be assessed. Fifth, we limited the assessment of sex differences to mortality, DCI, and functional outcome. Therefore, other outcomes, such as acute kidney injury, intensive care outcomes (i.e., need for tracheostomy, vasoactive drugs, renal replacement therapy, etc.), and subjective quality of life (QoL) measures, should also be considered. Regarding the number of limitations, our findings should be interpreted cautiously.

However, this review has also several strengths. First, according to our knowledge, this is the first systematic review that focuses at the same time on the association between sex and the outcomes of interest (mortality, DCI, and functional outcome) in patients with aSAH, providing the most up-to-date evidence that is based on a large number of patients included in our analysis. Second, we adopted strict inclusion criteria, particularly for the definition of DCI, despite the use of several terms/definitions across various studies, which posed a challenge. According to this, we excluded studies that did not provide a clear definition of DCI or those that involved asymptomatic vasospasm. In our opinion, this choice leads to results that are more homogeneous. Third, our analysis of functional outcome in terms of mRS only considered studies that defined a Modified Rankin Scale score of 0–2 as favorable, as recommended by the European Stroke Organization. As a result, our findings are more comparable and based on more homogeneous studies.

### 4.4. Conclusions

In conclusion, this study updates the available data on sex-related differences in patients with aSAH considering clinical outcomes. The results indicate that female patients are more likely to experience DCI after aSAH than males. However, there were no significant differences between the two sexes in terms of mortality and functional outcomes. Hence, as women represent a higher risk group for DCI, we suggest that they may require more intensive neuromonitoring during hospitalization.

We found that only a limited number of studies had sufficient gender data to include in our meta-analysis. Therefore, we suggest that more prospective studies with a focus on gender analysis are needed to obtain more robust results regarding sex differences in the clinical outcomes of aSAH patients.

## Figures and Tables

**Table 1:** Inclusion criteria: Scope of the literature review in the PICOS form. aSAH: aneurysmal subarachnoid hemorrhage; DCI: delayed cerebral ischemia.

Criteria	Definition
Population	− aSAH patients − ≥18 years old
Interventions	− None
Comparison	− Males vs. Females
Outcomes	− Mortality − DCI − Functional outcomes: modified Rankin Scale, Glasgow Outcome Scale, Glasgow Outcome Scale Extended
Study Design	− Randomized controlled trials − Prospective observational studies − Retrospective studies with more than ten patients − Clinical registries

## Data Availability

Data from previously published studies, in which informed consent was obtained, were retrieved and analyzed.

## Supplementary Materials

The following supporting information can be downloaded at: https://www.mdpi.com/article/10.3390/jcm13102781/s1, Table S1. Characteristics of the included studies for the mortality analysis. Table S2 Characteristics of the included studies for the delayed cerebral ischemia analysis. Table S3. Characteristics of the included studies for the functional outcome analysis.
