# Supplementary material for: Sex-Related Differences in Mortality, Delayed Cerebral Ischemia, and Functional Outcomes in Patients with Aneurysmal Subarachnoid Hemorrhage: A Systematic Review and Meta-Analysis
Source: J Clin Med. 2024 May 9;13(10):2781. doi: 10.3390/jcm13102781 (PMC11122382; doi:10.3390/jcm13102781)
Supplement: Supplementary file 1 [file jcm-13-02781-s001.zip › jcm-2992476-supplementary.pdf]

Supplementary Material

This supplementary material has been provided by the authors to give readers additional information about their work.

Article title: Sex-Related Differences in Mortality, Delayed Cerebral Ischemia, and Functional Outcomes in Patients with Aneurysmal Subarachnoid Hemorrhage: A Systematic Review and Meta-Analysis

Journal name: Journal of Clinical Medicine

Author names: Sarah Berli; Massimo Barbagallo; Emanuela Keller; Giuseppe Esposito; Alberto Pagnamenta; Giovanna Brandi

Affiliation and e-mail address of the corresponding author: Institute for Intensive Care Medicine, University Hospital Zurich, Switzerland, E-mail: giovanna.brandi@usz.ch

Supplementary Table S1 Characteristics of the included studies for the mortality analysis

Author	Year	Total (n)	Men (n)	Women (n)	Follow-up
Badjatia	2021	489	210	279	60 days
Bögli	2023	338	123	215	3 months
Crago	2015	99	37	62	12 months
Darkwah Oppong	2018	995	328	667	In-hospital mortality
De Marchis	2017	120	37	83	In-hospital mortality
Ding	2020	103	46	57	6 months
Festic	2013	318	114	204	In-hospital mortality
Fontana	2018	270	121	149	In-hospital mortality
Hamdan	2014	588	183	405	3 months
Kinter	2021	7271	2745	4526	In-hospital mortality
Lai	2019	294	67	227	6 months to 1 year
Lee S	2015	2849	1179	1670	In-hospital mortality
Macleod	2002	1023	401	622	In-and out-hospital mortality
Ramakrishna	2008	46	12	34	1 month
Rehmann	2020	577	179	398	In-hospital mortality
Schertz	2016	121	35	86	30 days
Stegmayr	2004	984	392	592	28 days
Tujjar	2017	202	78	124	ICU mortality
Virta	2022	1847	780	1067	12-months
**Total**		**18’534**	**7’067**	**11’467**	

Supplementary Table S2 Characteristics of the included studies for the delayed cerebral ischemia analysis

Author	Year	Total (n)	Men (n)	Women (n)
Abulhasan	2020	322	106	216
Badjatia	2012	50	17	33
Bakker	2007	321	90	231
Barletta	2013	42	21	21
Bjerkne Wenneberg	2020	55	15	40
Bögli	2023	343	125	218
Carrera	2009	441	123	318
Chen	2021	333	128	205
Crago	2015	99	37	62
Crobeddu	2012	292	99	193
Da Silva	2017	55	15	40
Darkwah Oppong	2018	935	329	606
De Marchis	2017	120	37	83
Duan	2018	504	209	295
Fan	2021	52	22	30
Fang	2019	702	264	438
Fischer	2019	30	9	21
Fontana	2018	270	121	149
Fukuda	2019	197	69	128
Germans	2018	6712	2306	4406
Guan	2016	24	9	15
Hamdan	2014	617	191	426
Heit	2018	16	5	11
Hirashima	2005	145	44	101
Hu	2022	109	36	73
Kasius	2010	91	24	67
Kaur	2021	149	41	108
Kawabata	2011	102	40	62
Ko	2011	160	58	102
Kozak	2016	434	176	258
Lai	2019	328	75	253
Lee H	2018	463	138	325
Megjhani	2021	388	115	273
Naraoka	2022	128	48	80
Neidert	2018	1321	468	853
Nguyen	2021	54	13	41
Oliveira Souza	2023	206	60	146
Park	2019	488	156	332
Platz	2017	504	168	336
Qureshi	2000	283	76	207
Raatikainen	2021	340	134	206
Rautalin	2022	1916	684	1232
Rehman	2020	544	169	375
Ritzenthaler	2021	349	122	227
Rowland	2017	22	8	14

Sanelli	2013	96	25	71
Saripalli	2021	175	51	124
Schembri	2021	90	27	63
Sorrentino	2023	119	30	89
Van der Steen	2019	369	117	252
Van Os	2020	582	167	415
Wu	2019	122	48	74
Yao	2017	237	110	127
Young	2016	579	216	363
Zhang	2021	439	135	304
**Total**		**23’864**	**8’126**	**15’738**

Supplementary Table S3 Characteristics of the included studies for the functional outcome analysis

Author	Year	Total (n)	Men (n)	Women (n)	Follow-up	Outcome
Bretz	2017	72	25	47	6 months	mRS
Crago	2015	65	23	42	3 months	mRS
Darkwah Oppong	2018	752	243	509	6 months	mRS
De Marchis	2017	120	37	83	6 months	mRS
Ding	2020	103	46	57	6 months	mRS
Güresir	2020	231	84	147	6 months	mRS
Hamdan	2014	588	183	405	3 months	mRS
Lai	2019	294	67	227	6 months to 1 year	mRS
Ois	2019	401	156	245	3 months	mRS
Unda	2020	177	52	125	12-18 months	mRS
Young	2016	579	216	363	3 months	mRS
Achrén	2022	108	53	55	3 months	GOS
Fontana	2018	270	121	149	3 months	GOS
Galea	2017	3341	1152	2189	hospital discharge	GOS
Pereira	2007	51	20	31	1 year	GOS
Qi	2021	49	15	34	30 days	GOS
Tujjar	2017	202	78	124	28 days	GOS
Bögli	2023	338	123	215	3 months	GOSE
**Total**		**7’739**	**2’694**	**5’045**		
